# Potential Novel Therapies for Neurodevelopmental Diseases Targeting Oxidative Stress

**DOI:** 10.1155/2021/6640206

**Published:** 2021-07-22

**Authors:** Alexandrina S. Curpan, Alina-Costina Luca, Alin Ciobica

**Affiliations:** ^1^Department of Biology, Faculty of Biology, “Alexandru Ioan Cuza” University of Iasi, Carol I Avenue, 20A, Iasi, Romania; ^2^“Grigore T. Popa” University of Medicine and Pharmacy, Strada Universitatii 16, Iasi, Romania; ^3^Department of Research, Faculty of Biology, “Alexandru Ioan Cuza” University of Iasi, Carol I Avenue, 20A, Iasi, Romania

## Abstract

Neurodevelopmental disorders are a category of diseases that is not yet fully understood. Due to their common traits and pathways, often it is difficult to differentiate between them based on their symptoms only. A series of hypotheses are trying to define their etiology, such as neuroinflammation, neurodegeneration, and immunology, but none have managed to explain their multifactorial manifestation. One feature that may link all theories is that of oxidative stress, with a redox imbalance as well as several other markers of oxidative damage (on lipids, proteins, and nucleic acids) being observed in both postmortem samples of the brain of patients with schizophrenia and autism spectrum disorders. However, the implication of oxidative stress in pathology is still distrustfully looked upon. For this purpose, in the current paper, we were interested in reviewing the implications of oxidative stress in these disorders as well as the impact of N-acetylcysteine on the oxidative status with a focus on the glutathione level and N-methyl-D-aspartate receptor. We were also interested in finding papers targeting the use of antioxidant properties of different plant extracts.

## 1. Introduction

Neurodevelopmental disorders (NDDs) are multifactorial conditions comprising a large spectrum of disabilities caused by some form of disruption in the normal developing of the brain [1]. These diseases are portrayed by impairments at the cognitive level and can be differentiated by a series of properties such as typical onset (childhood or puberty/early adulthood), clinical course (steady or remitting and relapsing), and origin (multifactorial or single cause), as well as using two theoretical approaches: *neuropsychological account* and *neuroconstructivism* to establish the differences between normal developing individuals and individuals with NDDs [2, 3].

Autism spectrum disorders (ASD) are NDDs with typical childhood onset characterized by fundamental debility in social behavior, absence or presence of communication difficulties, and restrictive and repetitive conducts [4]. Considering that ASDs are diagnosed in childhood, the most important role is played by pediatricians as they conduct ASD-specific screening (such as First Year Inventory, an instrument designed to identify infants at risk for ASDs, and the Communication and Symbolic Behavior Scales Developmental Profile—to receive early intervention even sooner) between 18 and 24 months old [5]. Screening methods applied in toddlers (14-30 months) have been proven to be more efficient than classic methods even in diagnosing early signs (like clinical judgement or caregiver concerns) suggesting that a partnership between pediatricians and autism specialists is more effective in systematic autism screening [6, 7]. One aspect has been correlated with the etiology of ASD—mitochondrial dysfunction [8]. Affected mitochondria whose function is damaged become the main source of reactive oxygen species, therefore causing an imbalance of the oxidative status of the cell. Oxidative stress can be described by damage at the molecular level affecting polyunsaturated fatty acids, proteins, and nucleic acids, but also by modified enzyme activity, modified fluidity, ion transport, and protein synthesis eventually leading to cell death [9]. Schizophrenia is a psychiatric disorder with puberty or early adulthood onset, characterized by 2 types of symptoms: positive (hallucinations and delusions) and negative (flattening effect or social withdrawal) paired with cognitive symptoms (impaired memory, attention, and executive functions) [10]. Schizophrenia has 2 hypotheses involving two types of receptors: D2 dopamine receptor (the target of the current established therapy) and N-methyl-D-aspartate receptor (an excitatory glutamatergic neurotransmitter) (NMDAR) [11]. NMDAR hypofunction together with redox imbalance (oxidative stress) might be the cause for defects in parvalbumin-positive interneurons (PVIs) described as an imbalance between excitation and inhibition and portrayed as responsible for schizophrenia' pathophysiology appearance and symptoms together with myelination defects [12].

Attention-deficit hyperactivity disorder (ADHD) is a common neurodevelopmental disorder characterized by attention problems, hyperactivity, and impulsivity. ADHD is usually diagnosed in childhood, but up to half of the cases continue in adulthood and are often associated with depression, anxiety, and substance abuse [13]. ADHD is believed to result from anomalies in the prefrontal cortex (PFC) which is a region deeply involved in executive functions including working memory, sustained attention, and decision-making; therefore, lesions of this area increase locomotor activity and impair cognitive processes. From a cellular point of view, the major constituents of PFC are glutamatergic pyramidal neurons and glutamatergic neurotransmission is a key factor in PFC-dependent functions. Glutamate signaling disruptions have been observed in several mental disorders, including schizophrenia and autism spectrum disorders [14].

## 2. Methods

### 2.1. Selection of Articles

We performed an initial literature search in January 2020 and included articles published after the year of 2000 with a focus on the most recent relevant ones. The articles were found by using the keywords under different combinations such as oxidative stress, schizophrenia, vegetal extract, glutathione, schizophrenia, N-acetyl cysteine neurodevelopmental disorders, and attention-deficit hyperactivity disorder. The search was performed by accessing MEDLINE, Hindawi, and Google Scholar databases.

**Figure 1:**
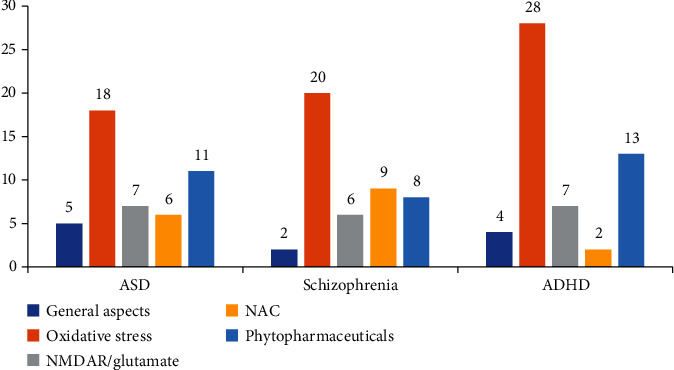
The division of the 73 articles selected based on the disorder (autism spectrum disorder (ASD), schizophrenia, and attention-deficit hyperactivity disorders (ADHD)) and +general aspects, +oxidative stress, +NMDAR (N-methyl-D-aspartate receptor/glutamate), +NAC (n-acetyl cysteine), and +phytochemicals.

Subsequently, articles were selected by the presence of the keywords in the title and abstract and their connection to neurodevelopmental disorders (or particularly to schizophrenia, autism spectrum disorders, and/or attention-deficit hyperactivity disorder) and oxidative stress, as well as potential therapies. We excluded articles related to disorders other than the previous mentioned ones, articles researching the effect of substances other than the ones of interest, and articles using herbal extracts or plants without assessing their antioxidant capacity. For the present article, we have selected a total of 114 articles, and their division based on the disorder (ASD, schizophrenia, ADHD) and +general aspects, +oxidative stress, +NMDAR/glutamate, +NAC, and +phytochemicals can be observed in Figure 1; from the total number of articles, 13 are about oxidative stress, and we have chosen to exclude this aspect from the figure.

### 2.2. Oxidative Stress

The brain is an organ with a very intense energetic activity, and the energy demand is fulfilled by oxidative phosphorylation of mitochondria, a process which may lead to the formation of reactive oxygen species—oxidative stress is a result of excessive formation of these species. In this case, protection mechanisms are compromised, reactive oxygen species start to accumulate, and neurons become susceptible to lesions [15].

Cellular redox status is a key player in various cellular functions and diseases. The accumulation of oxidants (such as reactive oxygen species, also known as ROS) has long been associated with oxidative stress [16]. Alongside ROS, there are several other reactive species such as reactive nitrogen species (RNS), reactive sulfur species (RSS), or reactive chlorine species (RCS) [17]. These species are produced as a product of oxygen-dependent cellular processes by cells. They have two possible roles—a positive one in which they are involved as signaling molecules in downstream reactions or an undesired role where they lead to oxidative stress. Oxidative stress results as an accumulation of oxidants inside the cell. Each cell has a specific threshold of endurance for this species; once that is exceeded, the cell and the organism itself cannot efficiently detoxify the reactive species [18]. It has also been observed that oxidative stress is linked with various physiological changes, implying damage to proteins, nucleic acids, lipids, and others. Therefore, cells must maintain a very precise balance between the benefiting effects of oxidants in signaling, immune responses, and biosynthesis and their downsides. Any sort of disturbance in this equilibrium can cause various dysfunctions and disorders [19].

Reactive oxygen species can be differentiated in two types following one criterion: whether they are free radicals or not. The most encountered free radicals are hydroxyl, superoxide, and nitric oxide, as they are produced during ionizing radiation or environmental toxicology reactions. As for nonradicals, the most well-known are hydrogen peroxide, singlet oxygen, ozone, and peroxynitrite. Nonradical species are less reactive from a chemical point view, but they can easily become radicals by reaction with macromolecules and cellular metabolites [20, 21].

A free radical is characterized by one or two unpaired electrons spinning on the peripheral orbital of the nucleus making it unstable and highly reactive. To acquire stability, the free radical is starting to extract electrons from the surrounding molecules, producing chain reactions leading to lesions [19, 21, 22]. In the oxidative stress state, the homeostasis tips towards the disease as a result of increasing reactive species and decreasing antioxidant defense [20].

During respiration processes, electrons migrate towards oxygen, mainly leading to unstable anionic superoxide radicals [23]. Superoxide anions increase under stress conditions such as intense physical exercise, certain medications, infections, and different states of disease. During normal metabolic processes, the human body produces more than 2 kg of superoxide anion per year [24]. Reduced free iron Fe^2+^ and copper Cu^1+^ participating in Fenton reactions produce radicals of the hydroxyl type as well as hydroxide anions, oxidizing the metals to Fe^3+^ and Cu^2+^. These anionic forms (hydroxyl type and hydroxide anions) can then oxidize H_2_O_2_ to form hydroperoxyl radicals (HOO^•^) and protons through a reduction reaction, so that new peroxidation reactions can occur in a cyclical manner [20]. One interesting aspect of reactive oxygen species is that they were proposed as a possible treatment for brain tumors due to their apoptotic effects. At all events, the ROS-based treatment is disputed due to their opposing effects: on the one side, they have a deleterious effect on cancerous cells; on the other side, they contribute to cellular proliferation and tumorigenesis [25].

Reactive oxygen species target oxygen sensible lipids such as glycolipids, cholesterol, and phospholipids, which can undergo lipid peroxidation. Interesting is that these lipid peroxides are beneficial to the cell in small quantities as they are used for cellular signaling via ligand-receptor interaction mediation using thiol reversible modifications [26]. When these lipid peroxides are generated in a nonspecific manner and they accumulate in membranes, they may lead to perturbations of the membrane bilayered structure affecting its permeability, plasticity, and as a result the ion gradient in cells. When these oxides are found in elevated quantities, 5-hydroxynonenal (4-HNE) and malondialdehyde (MDA) produce lesions to proteins, nucleic acids, and antioxidant molecules, eventually leading to the cell death [26].

Reactive oxygen species lead to DNA structure distortion and nucleotide alterations. To be more specific, the carbohydrate component of purine and pyrimidine nitrogen-containing nucleobases is susceptible to hydroxyl radical attack, a process which generates a large variety of labile oxidized derivatives [19]. As a consequence, DNA deterioration is often accompanied by mutagenesis and carcinogenesis [27]. It was observed that DNA and surrounding proteins form species by the cross-linking process binding guanine with lysine residues in a nonspecific manner, producing alterations in specific DNA-protein interactions and eventually leading to protein dysfunction and aggregation [28].

Proteins represent one of the main targets of ROS and RNS due to their high number of residues susceptible to oxidation. Their alteration may lead to misfolding, loss of function, and toxic aggregation [27]. The main pathway was excellently described by Stadtman and Levine [29]. Briefly, protein oxidation initiates chain reactions with a resulting toxic alkoxy radical, which either leads directly to peptide bond cleavage or reacts with the protonated form of the superoxide anion causing an increase in hydroxyl derivative production, hence affecting the secondary and tertiary structures of proteins. Moreover, most of the intermediate products of the chain reactions can individually convert adjacent amino acids in reactive radicals which could generate intra- and interprotein nonspecific covalent interactions; therefore, they have the potential to disrupt the structure and function of the oxidized protein.

Reactive oxygen species need to be strictly regulated and balanced to ensure the production of oxidants necessary for biogenesis and signaling purposes, without affecting self-macromolecules. Desired levels of ROS are acquired through the various multiple members of the redox homeostasis system, which is composed of antioxidants with small molecular weights (such as glutathione and vitamins A, C, and E) and detoxifying enzymes (such as thioredoxin, peroxiredoxin, and other oxidoreductases). The high diversity and abundance of the members of this system represent a prompt and specific response in the reestablishment of the possible cellular balance [19].

### 2.3. Oxidative Stress and Schizophrenia

Reactive oxygen species are involved in cell membrane pathology as described above and may play a role in schizophrenia and other psychiatric disorders as shown by a large body of evidence.

Altered mechanisms in schizophrenia may be a consequence of oxidative damage to lipids, proteins, and nucleic acids, defects which impact their neuronal functions. Although there are several theories regarding the etiopathogenesis of schizophrenia (such as neurodevelopment, neurodegeneration, immunology, inflammation, and infection), not even one has been confirmed beyond any doubt as none of them is capable of fully accounting all aspects of the disorder. One common feature of all these hypotheses is the implications of oxidative stress as an emerging important mechanism underlying several pathological processes [20].

Increasingly emerging evidence supports the cortical synaptic circuitry as the main deficit in schizophrenia, alongside other possible mechanisms affecting this circuit (such as dysregulation in neuronal apoptosis which may lead to an increase in cortical gray matter loss). These mechanisms start to be observed after the first onset of psychosis when antioxidant protection against oxidative stress is low [20].

Oxidative stress may also be observed at a biochemical level, especially in the dopaminergic system due to the excessive production of free radicals leading to oxidative damage in the brain structure. This hypothesis has been reported by several studies supporting the general theory of a progressive route and a neurodegenerative origin of this disorder. Additionally, neuroinflammation cannot be put aside as an overproduction of prostaglandins from arachidonic acid and a production of proinflammatory cytokines (IL-6) have been observed. The synthesis of prostaglandins is fulfilled by cyclooxygenase-2 (COX -2), whose levels have also been reported to be elevated [20].

Despite the numerous biomarkers for oxidative stress, one that is notably important is glutathione (GSH); it can be used to determine oxidative damage to lipids, proteins, and DNA in both central and peripheral areas with a deficit in antioxidant defense. One key aspect is that the changes due to this imbalance are not restricted to the brain area but can also be observed in body fluids (such as blood) and peripheral cells (plasma, erythrocytes, and blood platelets). To assess the impact of oxidative stress on schizophrenia patients, animal models have been used. By means of these experiments, elevated levels of lipid peroxidation products have been observed and altered proteins and amino acids (e.g., estimated by the level of generated carbonyl groups or protein peroxides), 3-nitrotyrosine, and DNA damage products (hydroxy guanosine, telomere shortening). Regarding fatty acids, to be more precise, polyunsaturated fatty acids (PUFAs)—components of membrane phospholipids, whose dysregulation has been reported to some extent—had reduced levels. Impairment of membrane phospholipids by means of free radicals leads to disturbances in signal transduction and may be also linked to functional changes regarding neurotransmitters—especially glutamatergic and serotonergic [20].

Therefore, dysregulation in these macromolecules may be associated with schizophrenia pathology.

### 2.4. Oxidative Stress and Autism Spectrum Disorders

Similar to schizophrenia, studies on postmortem brain samples acquired from individuals affected by autism spectrum disorders (ASD) indicate the involvement of oxidative stress in the pathology of this disorder.

Several studies have reported damage of oxidative nature to lipids, proteins, and nucleic acids and a decreased level of the major cellular antioxidant—GSH. Two studies using, respectively, 10 [30] and 15 [31] individuals and age-matched controls have made one common observation—decreased levels of GSH/GSSG in the cerebellum, with one also observed in the temporal cortex and the other in the Brodmann area. Interesting is that these findings are not correlated with age, but more observed as a chronic condition [30–32].

Damage to lipids is represented as significantly higher levels of hydroperoxides of lipid origin in the temporal cortex and cerebellum and mitochondrial dysfunction in the same areas [32].

When it comes to proteins, oxidation at this level was observed by measurements of 3-nitrotyrosine (3NT) levels and was reported to be elevated in the cerebellum and correlated with increased neurotrophin-3 in the same brain area. This protein has a critical role in normal brain growth and differentiation, and its increase may be related to oxidative stress [31, 32].

In studies conducted for identifying oxidative damage to nucleic acids, the level of two biomarkers was pursued—8-oxo-deoxyguanosine [33] and 8-hydroxydeoxyguanosine [34] both for DNA—and was found to be elevated in the temporal lobe and in the cerebellum, respectively, while the one for RNA—8-hydroxyguanosine [35]—was higher in the frontal cortex when compared to controls [32].

Mitochondrial dysfunction was also observed in postmortem brain samples of individuals suffering from ASD under the form of decreased activity of the electron transport chain (ETC) complex and tricarboxylic acid (TCA) cycle enzyme, as well as discrepancies in gene expression [32].

### 2.5. Oxidative Stress and Attention-Deficit Hyperactivity Disorder

In the case of ADHD, the hypothesis of oxidative stress as an etiological factor for developing the disorder only comes from studies illustrating treating antioxidant substance efficiency. Interestingly enough, this particular study has found no significant difference in terms of antioxidant activity but found a slight increase in oxidative stress [36]. This might be explained by the fact that the ADHD patients have a defective response to oxidative stress in spite of normal production of antioxidants [37].

ADHD is characterized by a dopamine deficiency, and one study in particular has observed that the striatal dopamine release is suppressed by the elevated hydrogen peroxide levels they found in their sample [38]. Even if dopamine possesses antioxidant and free scavenging properties, it can also be easily oxidized generating highly reactive metabolites which further lead to mitochondrial dysfunction and oxidative stress [37]. Zinc is a known cofactor in reactions involved in antioxidant defense reactions, and a study found lower plasma levels of zinc in ADHD patients supporting the idea of oxidative stress involvement in ADHD pathophysiology [39, 40].

It also seems that nitrosative stress also has a role in ADHD [41]. One study has reported nitric oxide/melatonin and malondialdehyde/melatonin ratios lower in ADHD children [42] whereas the study of Selek et al. identified increased levels of NO [43]. They also found a low SOD activity in adults with ADHD which can be associated with oxidative stress, but no difference in children [44]. The authors have also observed altered activity of glutathione peroxidase and increased oxidative DNA damage. These findings suggest that patients with ADHD have normal levels of antioxidant production, but their response is flawed.

### 2.6. Glutathione

An imbalance between the levels of prooxidants and antioxidants produces oxidative stress which in return leads to macromolecular impairment. The high lipid content, the metabolic rate, and the nonregenerative nature of CNS neurons make it challenging to maintain the redox imbalance in the brain. Even the smallest perturbations to the redox balance during development can affect the signaling pathways and processes involved in neurogenesis and neuronal differentiation. The antioxidant systems function to maintain the redox balance by supplying reducing equivalents (electrons) to electrophilic xenobiotics, ROS, and proteins. The antioxidant glutathione (GSH) has special relevance to schizophrenia pathophysiology [11].

Studies conducted on schizophrenia patients revealed elevated levels of protein and lipid oxidation, measured in blood, cerebrospinal fluid, and postmortem tissue, as well as altered levels of CSF superoxide dismutase-1 (SOD1) and plasma antioxidants, where GSH levels have been shown to be lower in chronic schizophrenia as demonstrated by proteomic studies. Likewise, animal studies suggest that GSH deficits and oxidative stress in the developing brain are sufficient to induce schizophrenia-like behavior by a deficiency in the glutamate-cysteine ligase (GCL) regulatory subunit G*clm*. Pharmacological depletion of brain GSH using a GCL inhibitor induces similar sensory and cognitive disturbances [11].

Growing evidence shows that redox imbalance and oxidative stress are key factors in the physiological pathology of schizophrenia. Redox imbalance can be attained by several pathways affecting both enzymatic and nonenzymatic antioxidant systems. One of the pathways is the deficit in glutathione (GSH), which is the main antioxidant and redox regulator in the brain. By direct measurements of cerebrospinal fluid or via MR spectroscopy in the frontal lobe, GSH has been shown to be decreased in some patients' brains [45].

### 2.7. N-Methyl-D-aspartate Receptor

N-Methyl-D-aspartate receptors (NMDARs) are glutamate receptors that act as cation-passing channels that have a key role in the CNS [11]. The role of NMDAR in learning and memory seems to have a well-established position throughout animals including humans, and administration of NMDAR antagonists has been validated as a cause for learning impairment [46].

Due to observations made after the administration of the dissociative anesthetics, phencyclidine and ketamine, to healthy subjects, mimicking the primary symptoms of schizophrenia coupled with the fact that these two compounds are NMDAR antagonists brought forth the NMDAR hypofunction hypothesis. By treating healthy subjects with NMDAR antagonists, indicators of sensory dysfunction in patients with schizophrenia can be seen—such as mismatch negativity (MMN) and changes in auditory and visual event-related potentials. Another activity that may be attributable to NMDAR hypofunction is hyperglutamatergic activity. Genetic analysis additionally points towards a disrupted NMDAR signaling disturbed in schizophrenia [11].

A series of studies performed on adult rodents by using NMDAR antagonists revealed schizophrenia-like behaviors, including deficits in attention, learning, memory, and sensory gating [11]. NMDAR dysfunction has also been suggested as a hypothesis for ADHD after numerous studies [47].

In studies targeting prenatal risk factors for schizophrenia, administration of NMDAR antagonists during the equivalent period in rodents induces long-term behavioral and cognitive disruptions that are relevant to the schizophrenia phenotype, disturbances that prolong into adolescence and adulthood. Furthermore, severe NMDAR blockade induces forebrain apoptosis in rats if it occurs between the first and third postnatal weeks [11].

An increasing body of evidence points towards a possible reciprocal link between NMDAR hypofunction and redox imbalance, as both are mechanisms underlying selective impairment in PVI function and the disturbance in behavior and cognition [11].

The NMDAR is regulated by the redox state as it possesses pairs of redox-sensitive cysteine residues whose disulfide bond formation decreases NMDAR currents, while an overlapping group of cysteine residues is subject to inhibitory S-nitrosylation, which facilitates disulfide bond formation. Therefore, the redox balance controls NMDAR activity through GSH, and the other way around is also valid by the means that NMDAR hypofunction leads to cortical oxidative stress and GSH deficits [11, 48].

Although the coupling of synaptic NMDAR activity to the control of antioxidant defenses is still at the level of speculation, this coupling may be an adaptive mechanism to connect neuronal antioxidant defenses to the electrical and metabolical needs of an active neuron [48]. NMDAR hypofunction also contributes to oxidative stress by decreasing the activity of cortical interneurons. NMDAR antagonism probably induces a hyperglutamatergic state [11, 49].

Neurons have a comparatively weak inherent antioxidant defense, hence relying on astrocytes to provide external support. Astrocytes, in response to oxidative stress, increase the production and release of GSH, which will be broken down and used for neuronal GSH production. Disregarding the continuously growing body of evidence linking NMDAR hypofunction and redox imbalance, different genetic and environmental factors can assemble in a similar pathological outcome of schizophrenia-like phenotypes [11, 50].

Besides oxidative stress, another process which may be involved in the etiology of schizophrenia with a considerable amount of evidence is neuroinflammation. Studies conducted on mice showed that maternal infection is sufficient to induce long-term prepulse changes in the offspring—changes that could be due to inflammation. Inflammation is likely to be a risk factor in the effects of NMDAR hypofunction and oxidative stress [11].

A new approach regarding the possible treatments for schizophrenia and/or neurodevelopmental disorders is antioxidant therapies [11].

## 3. Redox Diagnostic Biomarkers

### 3.1. ASD and Diagnostic Biomarkers

ASD is associated with dysregulations involving methylation processes and redox metabolism. Abnormalities in these processes lead to oxidative damage targeting lipid, proteins, and nucleic acid making the assessment of pro- and antioxidant levels a potential diagnostic tool allowing for early intervention in order to correctly diagnose or reduce the severity of symptoms. In a pediatric setting, several different analyses have been developed in order to better characterize ASD and have a higher accuracy of classifying ASD. For example, one analysis in particular (Fisher discriminant analysis) is based on folate-related metabolism markers, and it is capable of differentiating ASD from healthy individuals with a 97% accuracy [51]. Another potential analysis method is constituted by the Autism Biomarkers Consortium for Clinical Trials which uses a neurophysiological biomarker, N170, response to face stimuli, reporting delayed and lower amplitude responses in children, adolescents, adults with ASD, and children and adults with Asperger's syndrome. However, it has not been used yet for differentiating between control groups and ASD groups [52]. The Children's Autism Metabolome Project (CAMP) has successfully identified a subgroup of ASD that presents a dysregulated branched chain amino acid metabolism with a 96.3% specificity and a 93.5% positive predictive value, but it can only identify this specific subgroup [53].

One of the primary oxidative stress markers is glutathione (GSH), both in its free form and in the oxidized disulfide form (GSSG). GSH is capable of converting hydrogen peroxide into water, reaction catalyzed by glutathione peroxidase (GPx). Studies have reported that total glutathione and free reduced glutathione levels significantly reduced in autistic patients, whereas the oxidized form was found to be significantly increased [54, 55]. A reduced SAM/SAH ratio is known to be associated with hypomethylation of DNA, RNA, proteins, and phospholipids as one study illustrated a SAM/SAH ratio reduced in ASD associated with a decrease in cysteine [54]. Abnormalities in redox metabolism as measured by alternations in glutathione redox status were reported by several case-control studies using immune cells, lymphoblastoid cell line, and postmortem brain tissue, and dysregulation of methionine and cysteine has also been observed. Children with autism exhibited a significant decreased extracellular concentration of GSH and GSH/GSSG and increased concentration of GSSG. Low glutathione redox status has also been associated with the pathophysiology of schizophrenia [56].

### 3.2. ADHD and Diagnostic Biomarkers

One study conducted on spontaneously hypertensive rats (established model animal of ADHD) has reported significant increases in serum and/or tissue concentrations of cytokines and oxidative stress markers in juvenile ADHD model animal suggesting a correlation between neurological and immune systems in ADHD pathogenesis; however, there was no difference between the animal model and the control group of 10 weeks old [57].

One study has found significantly higher levels of GSH in ADHD children compared to controls, and they have also reported differences based on sex, with disulfide levels being higher in males suggesting that thiol/disulfide homeostasis is abnormal in children [58, 59], results similar to another study, whereas Oztop et al. [60] did not find differences and Guney et al. have observed lower plasma thiol in children [61] and adults, alongside lower 8-hydroxy-2-deoxyguanosine. Results over catalase levels are also contradictory with studies reporting no difference between the control group and ADHD group [44, 62] and other observing lower catalase activity [63]. A similar situation is for MDA levels with studies reporting lower levels of lipid peroxidation measured through this biomarker [60] and higher levels [44, 64–66].

Iron deficiency has been suggested as a possible factor contributing to the etiology of ADHD in children since it could lead to oxidative damage, as two studies have noted lower Cu-Zn-dependent SOD [67, 68] .

One study performed with adults with ADHD has observed no significant differences in terms of total antioxidant status, total oxidant status, and oxidative stress index [69]. All of these results make the use of oxidative stress biomarkers difficult, and it suggests that ADHD patients are able to produce normal levels of antioxidants, but their response is not sufficient [37].

### 3.3. Schizophrenia and Diagnostic Biomarkers

Out of the three disorders chosen for this article, schizophrenia has the most complete and clear oxidative stress picture. However, an interesting study conducted on schizophrenia patients drug-free has reported reduced plasma GSH and total thiol but significantly increased MDA and GPx [70]. Another one using first-episode schizophrenia patients and chronic schizophrenia patients reported significantly higher plasma MDA levels [71], whereas another study noted that in the case of first-episode psychosis, the levels of SOD and CAT were 40% lower, reduction that can be ameliorated by antipsychotic administration [72]. Elevated MDA levels are confirmed by other studies as well [73], alongside NO [74–77] and decreased levels of GSH, SOD [74, 75, 77, 78], reduced catalase, GPx, glutathione reductase, total antioxidant status, and lower peroxidation [75, 76, 79, 80]. One study has observed increased cellular damage, but not significant; however, they have noted a significant higher DNA damage in males compared to the female group [81] and another study observed elevated serum prooxidant balance in patients with schizophrenia [82].

All these results are suggesting that oxidative stress biomarkers are a direction with great potential when it comes to schizophrenia diagnostic, opening an even broader area of possible therapies.

## 4. Current Potential Therapies

### 4.1. N-Acetylcysteine

N-Acetylcysteine (NAC) (molecular formula: C5H9NO3S) is an acetylated derivative of cysteine, an amino acid containing sulfur. N-Acetylcysteine has been used as a prodrug in the clinical treatment of paracetamol overdose for over 30 years as it is an antioxidant precursor to glutathione (*γ*-glutamyl cysteinyl glycine; GSH). As more is understood about the actions of NAC, the clinical applications have also broadened, with now being used as a mucolytic in the treatment of chronic obstructive pulmonary disease, cystic fibrosis, and contrast-induced nephropathy and in the treatment of HIV. N-Acetylcysteine is widely available in many countries, as a nutritional supplement for brain function. Increasingly, it is being explored as an adjunctive therapy for many psychiatric conditions. Specific to brain disorders, NAC has been trialed with some efficacy in patients with Alzheimer disease [83, 84].

N-Acetylcysteine (NAC) influence on glutamatergic neurotransmission has been established after numerous attempts to correlate it with symptom severity reduction. [12].

The use of NAC in correcting GSH levels is well approved. Glutathione is the primary endogenous antioxidant and through direct and indirect scavenging has the ability to neutralize reactive oxygen and nitrogen species, being responsible for maintaining the oxidative balance in the cell. The direct method is a cycle starting with the formation and breakdown of adducts catalyzed by glutathione peroxidase (GPx) in a NADPH-dependent reaction, continuing with the resulting oxidized glutathione being reduced by glutathione reductase to restart the cycle.

Astrocytes contain much higher levels of GSH and then neuronal cells and release GSH into the extracellular space, which is then broken down by *γ*-glutamyl transpeptidase to a cysteine-glycine dipeptide (which will be hydrolyzed to glycine and cysteine) and glutamate with all 3 amino acids becoming available for neuronal GSH synthesis. Neuronal GSH production is believed to be primarily mediated by astrocytic GSH release, and astrocytic GSH production is rate-limited by cysteine and the enzyme glutamate-cysteine ligase [84].

Providing cysteine for GSH production is not the only property of NAC; it has been shown that it also has the ability to directly scavenge hydroxyl radicals and hypochlorous acid [84].

Oral NAC administration increases cysteine levels, ultimately leading to the increase in plasma GSH, and can also penetrate the blood-brain barrier (BBB), therefore raising the brain GSH levels unlike oral administration of GSH alone and/or L-cysteine which has little effect due to first-pass metabolism and the poor capacity to penetrate the BBB. Literature findings illustrate an increased level of brain glutathione after N-acetylcysteine was administrated in its oral form in animal models [83, 84].

As previously stated, besides oxidative stress, neuroinflammation might be another process involved in the pathology of schizophrenia. NAC use has the potential to modulate the pathways involved in inflammation, therefore increasing its benefits in the field of psychiatry. The mechanism underlying NAC action directed at neuroinflammation is through inflammatory cytokines having as a result the modulation of psychiatric symptoms. This may be directly associated with the inflammatory pathway or working through oxidative processes associated with inflammation [83, 84].

N-Acetylcysteine regulates glutamate via the cysteine glutamate antiporter (system Xc-) and glial glutamate transporter (GLT1), both essential components of glutamate homeostasis. At the level of this antiporter system, an exchange occurs between extracellular glutamate and intracellular cysteine in a 1 : 1 ratio, promoting the activation of mGlu2/3 receptors and inhibiting the presynaptic release of glutamate, helping to regulate glutamatergic neurotransmission through negative feedback by binding glutamate to N-methyl-D-aspartate receptors. Reduced expression of the system Xc- and GLT1 is associated with higher levels of synaptic glutamate transmission, decreased tone on mGlu2/3 receptors, reinstatement of drug-seeking behavior associated with addiction withdrawal, and the pathology of repetitive behaviors [12, 83]

In schizophrenia, increased dopaminergic metabolism in the striatum has been reported. This hyperdopaminergic state has been shown to inversely correlate with hypodopaminergia in the prefrontal cortex. These changes are believed to mediate alterations in executive function and many of the positive symptoms of the disorder. In populations with schizophrenia, dysfunction in glutamate metabolism and decreased glutamate levels in the prefrontal cortex have been reported [84].

Several clinical studies were conducted to study the effect of NAC on glutathione and redox dysregulation as a base for oxidative stress in various schizophrenia patients.

Initial clinical studies involving NAC supplementation in schizophrenia focused on glutathione and redox dysregulation as an origin for oxidative stress in schizophrenia and the impact of NAC supplementation; these studies were able to characterize the different associations between NAC and neurotransmitter pathways (glutamate receptors) with finding strongly suggesting that NAC supplementation improved NMDAR activity [12]. Even if more studies are required to support the use of NAC as an adjuvant therapy for ADHD, one study has shown the ability of NAC to reduce the ADHD symptoms in patients with systemic lupus and even block the autoimmune inflammatory system and block the production of IL-18 and IL-1*β* [85].

One trait is evident throughout the wide spectrum of psychiatric disorders, specifically neurotransmitter dysregulation. Supplementation with N-acetylcysteine portrays a rising therapeutic alternative as it has been shown to abate the dysregulation of the two neurotransmitter pathways involved in the etiology of schizophrenia as well as increasing glutathione levels [83] as shown in Table 1.

### 4.2. Herbal Extracts and Plants with Antioxidant Properties

As days are passing by, increasing scientific breakthroughs hit the pages of a journal, especially when talking about alternative medicine. The use of plants to treat different disorders has started to gain more ground as their beneficial properties become more well known. This also applies for diseases that have an oxidative origin or are influenced by it.

Sulforaphane, a compound found in great quantities in the seeds of numerous cruciferous plants, has shown the ability to raise glutathione levels and even fight oxidative stress in animal models as one pilot study suggests [94].

Polyphenols are micronutrients found in plants that are rich in antioxidants and have been associated with a lower incidence of psychiatric disorders; through their chemical structure, they are able to cross the blood-brain barrier and have been associated with neuronal protection [95]. From this class of compounds, flavonoids have been suggested to have an antioxidant and redox modulating ability. *In vitro* experiments have shown the great antioxidant potential of polyphenols through mechanisms such as radical scavenging where the position and number of -OH groups determine the strength of the reaction [96]. One study conducted on ADHD patients testing the effects of polyphenolic compounds from *Ginkgo biloba* at a 80-120 mg dose have illustrated no significant improvements [97], whereas another study using a double dose illustrated significant improvement in terms of ADHD symptoms and brain electrical activity [98]. *Ginkgo biloba* administered to schizophrenia patients has illustrated significant improvement in terms of positive symptoms, general psychopathology, and adverse effects of antipsychotics; however, no significant effect on negative symptoms has been noted [99].

*Hypericum perforatum* is an herbaceous perennial plant that has been long used in traditional medicine and is rich in flavonoids, phenolic acids, and tannins. One double-blind, randomized, placebo study in particular has observed positive results in ADHD patients after an 8-week treatment period [100].

Alongside *Ginkgo biloba* and *Hypericum* extract, lavender oil has also been shown to be efficient in treating anxiety and depression [101], whereas *Salvia officinalis* and *Rosmarinus officinalis* have cognitive-enhancing potential [102] and ginseng extract is believed to be able to improve attention deficit, cognition, and mental health [103].

Lemon balm in association with *Valeriana officinalis* root has been deemed effective in the treatment of ADHD in children by improving concentration, hyperactivity, and impulsiveness, whereas *Valeriana* alone helps with sleeping problems [104, 105].

Green tea extract showed anxiolytic and sedative effects possibly due to neuromodulation of dopamine and serotonin in certain brain areas; however, it has been observed that higher doses should not be associated with drugs metabolized by the CYP450 isoenzyme family, which is the case for the psychotropic drugs used in the treatment of ADHD, ASD, and schizophrenia as it leads to hepatotoxicity [95].

*Curcuma longa* is an Indian spice known for its protection effects on neurodegenerative disease and neuropsychiatric disorders with the main curcuminoid being curcumin. Curcumin is able to increase glutathione and reduce inflammatory components and mitochondrial dysfunction as well as oxidative/nitrosative stress, and several studies on animal models of autism have reported significant amelioration of symptoms and restoration of all changes related to the ASD phenotype in a dose-dependent manner [106–108].

Pycnogenol, a standardized extract from the bark of *Pinus pinaster*/*Pinus maritima*, is a potent antioxidant compound capable of reducing the free radicals through its rich composition in phenolic acids, polyphenols, and flavonoids. Several studies have reported significant improvements of ADHD symptoms in children; however, they also observe relapsed once the treatment was stopped. Pycnogenol has been observed to decrease dopamine levels and oxidative DNA damage and improve the GSH/GSSG ratio indicating that this extract might inhibit oxidative stress through normalizing catecholamine levels in children [109].

Piperine is the alkaloid responsible for the pungency of black pepper and has neuroprotective effects on glutamate suggesting that oxidative stress alterations might be reversed following treatment, as some studies report restored motor deficit and decreased reorientation time as well as restoration of cerebellum integrity by decreasing the number of Purkinje cells in ASD [110, 111].

Ziprasidone is an atypical antipsychotic drug used in the treatment of schizophrenia and induces plasma lipid peroxidation. One study has observed that this side effect could be inhibited by the polyphenols contained by *Aronia melanocarpa* berries [112].

## 5. Conclusions

The amount of evidence supporting the role of oxidative stress in the progression of neurodevelopmental disorders seems to increase and becomes more visible as new evidence is piling up. As these disorders have a very complicated origin and high comorbidity, a trait that could tie together all symptoms and hypotheses has the potential to shed some light on how therapies should be approached in the future.

Oxidative stress has always been considered the “black hole” of science as it seems to be involved in a large repertoire of disorders, without having a focus. As all areas of science have evolved enormously compared with the times when traditional medicine was used, in the current times, we can use them as therapies based on their phytochemical components; therefore, they have a big potential as targeted drugs and future studies should focus on the antioxidant properties of plant extracts.

N-Acetylcysteine as a repurposed drug for several times has the potential to improve negative symptoms of schizophrenia, when compared to antipsychotic effects, and to improve irritability and social traits in autism spectrum disorders individuals; hence, future studies should be realized on larger scales and in association with different types of medications; it would be also interesting to test its abilities right after the onset of these disorders.

## Figures and Tables

**Table 1:** Antioxidant properties of N-acetylcysteine in neurodevelopmental disorders.

Authors	Model	Number of participants	Doses	Results
[86] (substance abuse)	Monkeys (*Macaca mulatta*)	5 young males	(i) 150 mg/kg/h, i.v. over 30 min and 12 mg/kg/h, i.v. over 9 hours	(i) May attenuate the decrease in dopamine transporters after methamphetamine adm.
[87] (propionic acid- (PPA-) induced biochemical autistic features)	Rats (western albino)	28 young males divided into 4 groups (control, PPA treated, NAC → PPA (protective), PPA → NAC (therapeutic))	2–250 mg/kg/day PPA 3 d 3–50 mg/kg/day NAC 1 w → PPA 3 d 4—toxic dose PPA → same dose NAC	(i) NAC successfully defied the oxidative stress induced by propionic acid administration
[88] (double-blind placebo-controlled schizophrenia patients (<60% medicated with clozapine)	Human	150	1000 mg bidaily (6 months)	(i) Improved negative symptoms, but the improvements were lost 1 month after the end of the trial (ii) Side effect: gastrointestinal
[89] Double-blind (schizophrenia)	Human	11	2000 mg/day (oral) (8 weeks)	(i) Significant improvements regarding mismatch negativity and plasma glutathione concentration
[90] (treatment-resistant schizophrenia)	Human	1 female	600 mg/day (oral)+usual medication	↓ Positive and Negative Syndrome Scale (PANSS) ↓ Clinical Global Impression
[91] (randomized control trial, early psychosis)	Human	63 (32 NAC, 31 placebo)	2700 mg/day effervescent NAC (1800 mg in the morning, 900 evening, 6 months)	↑ by 23% brain GSH levels in the medial prefrontal cortex (i) Improvement in positive symptoms, cognition, level auditory processing, and white matter diffusion
[92] (double-blind randomized controlled pilot trial, autism)	Human	33 (31 males, 2 females, ages 3.2-10.7 years)	900 mg/day (4 weeks) → 900 mg/twice per day (4 weeks) → 900 mg/thrice per day (4 weeks) (oral)	(i) NAC groups presented significant improvements on ABC-irritability subscale (ii) Side effect: gastrointestinal
[93] (randomized placebo-controlled trial, autism)	Human	31 in the beginning (NAC 16, placebo 15—3 lost to follow-up, 3 left the trial) (4-12 years)	(i) Doses ranging from 33.6 to 64.3 mg/kg (12 weeks)	(i) GSH level significantly higher in the NAC group (*p* < 0.05), increased glutathione disulfide (*p* = 0.09) (ii) No significant impact on social impairment
